# Characterization of the aurantimycin biosynthetic gene cluster and enhancing its production by manipulating two pathway-specific activators in *Streptomyces aurantiacus* JA 4570

**DOI:** 10.1186/s12934-016-0559-7

**Published:** 2016-09-21

**Authors:** Houyuan Zhao, Liang Wang, Dan Wan, Jianzhao Qi, Rong Gong, Zixin Deng, Wenqing Chen

**Affiliations:** 1 Key Laboratory of Combinatorial Biosynthesis and Drug Discovery, Ministry of Education, School of Pharmaceutical Sciences, Wuhan University, Wuhan, 430071 China; 2 State Key Laboratory of Microbial Metabolism, School of Life Sciences & Biotechnology, Shanghai Jiao Tong University, Shanghai, 200030 China

**Keywords:** Aurantimycin, Gene cluster, Pathway-specific activators, *Streptomyces*, Biosynthesis

## Abstract

**Background:**

Aurantimycin (ATM), produced by *Streptomyces aurantiacus* JA 4570, is a potent antimicrobial and antitumor antibiotic. Although the chemical structure of ATM is highly distinctive and features a cyclohexadepsipeptide scaffold attached with a C_14_ acyl side chain, little is known about its biosynthetic pathway and regulatory mechanism.

**Results:**

In this work, we report the identification and characterization of the ATM biosynthetic gene cluster from *S. aurantiacus* JA 4570. Targeted inactivation of *artG*, coding for a NRPS enzyme, completely abolished ATM production, thereof demonstrating the target gene cluster (*art*) is responsible for ATM biosynthesis. Moreover, four NRPS adenylation (A) domains including a freestanding enzyme ArtC have been characterized in vitro, whose substrate specificities are consistent with in silico analysis. Further genetic analysis of the two regulatory genes *artB* and *artX* unambiguously suggested both of them play positive roles in ATM biosynthesis, and ATM-A production was thus rationally enhanced to about 2.5 fold via tandem overexpression of *artB* and *artX* in *S. aurantiacus* JA 4570.

**Conclusions:**

These results will provide the basis for the understanding of precise mechanisms for ATM biosynthesis, and open the way for both rational construction of high-production ATM producer and orient-directed generation of designer ATM derivatives via synthetic biology strategies.

**Electronic supplementary material:**

The online version of this article (doi:10.1186/s12934-016-0559-7) contains supplementary material, which is available to authorized users.

## Background

Aurantimycin (ATM), produced by *Streptomyces aurantiacus* JA 4570 (*S. aurantiacus* hereafter), was isolated in 1994 in the process of screening for new ionophoric compounds [1]. ATM exhibited high bioactivity against the gram-positive bacteria including *Bacillus subtilis* ATCC 6633 (MIC 0.013 µg/mL) and *Staphylococcus aureus* 285 (MIC 0.013 µg/mL), but gram-negative bacteria and fungi were not susceptible to this antibiotic [1]. Moreover, ATM showed cytotoxic effects against L-929 mouse fibroblast cells causing a sudden change from nontoxic to lethal concentrations ranging from 3 to 12 ng/mL [1].

**Fig. 1:**
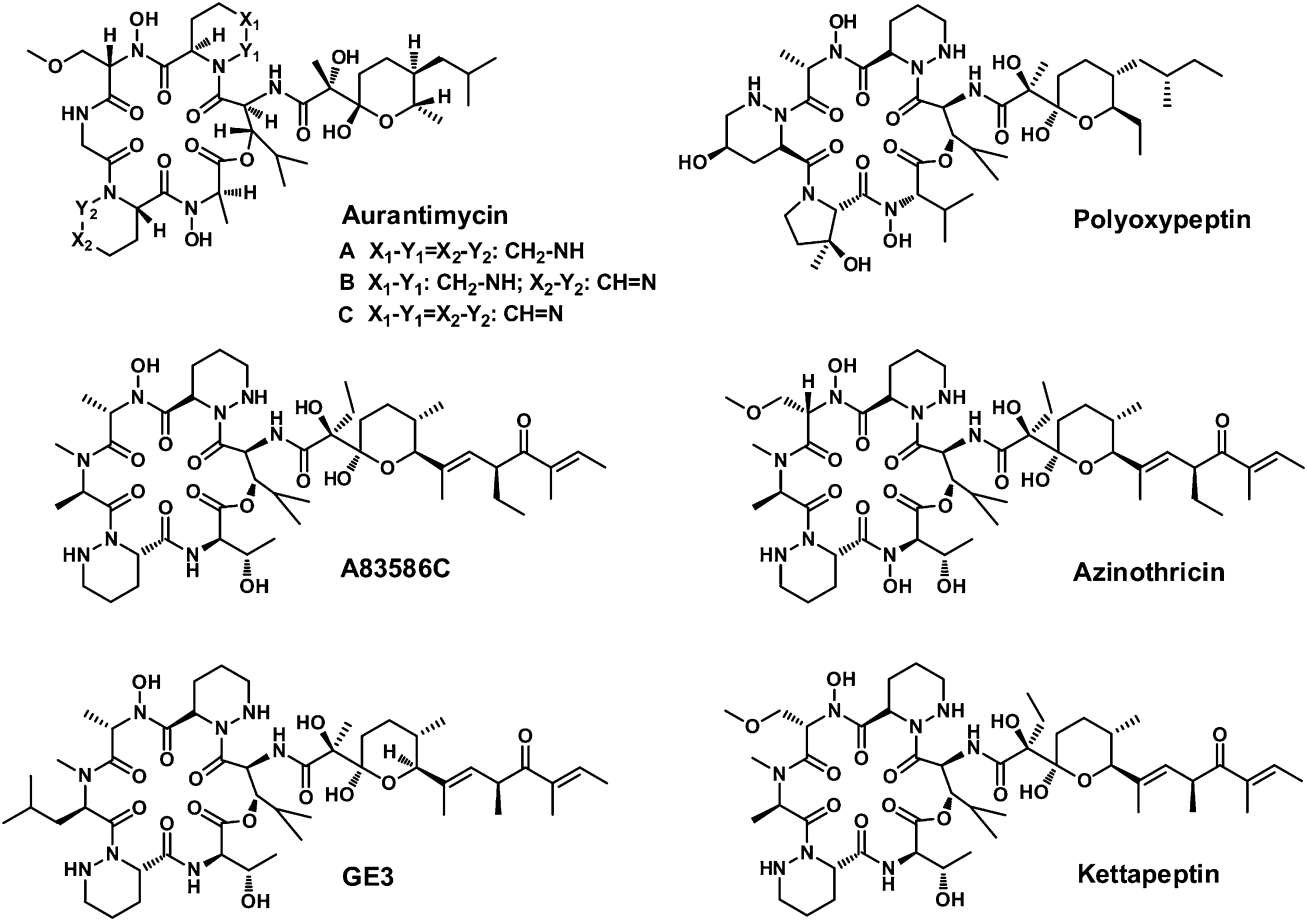
Chemical structures of ATM, and other related azinothricin-family antibiotics

ATM features a 19 membered cyclohexadepsipeptide core scaffold attached by a C_14_ acyl side chain. Structurally, different ATM components share a distinctive structural feature with a tetrahydropyranyl-propionic acid acyl side chain linked to *2S, 3S*-hydroxyleucine residue of the core scaffold via an exocyclic amide bond (Fig. 1) but differing in the extent of unsaturation of two opposite piperazic acid moieties. Notably, despite the unique structure and diverse distribution of such moiety in natural products, the precise mechanism for its biosynthesis remained unknown and confused biochemists for decades. In addition, ATM also contains other nonproteinogenic amino acids, such as *N*-hydroxy-L-alanine, *N*-hydroxy-L-serine, *O*-methyl-*N*-hydroxy-L-serine, and a proteinogenic amino acid glycine.

Structurally-related natural products, in the past decades, have been continuously discovered from Actinomycetes including A83586C [2], azinothricin [3], citropeptin [4], diperamycin [5], kettapeptin [6], IC101 [7], L-156,602 [8], pipalamycin [9], polyoxypeptin [10] and variapeptin [11] (Fig. 1). Such group of secondary metabolites was designated as “azinothricin family” antibiotics as azinothricin, isolated from *Streptomyces* sp. X-1950 in 1986, was identified as the first member, and the biosynthetic gene cluster of polyoxypeptin has been recently reported by Du et al. [12]. Attractively, this family of secondary metabolites exhibited diverse biological activities, including potent antibacterial, antitumor [13, 14], anti-inflammatory activities [15], and acceleration of wound healing [16]. The majority of azinothricin-family members showed powerful antitumor effects [17]. For example, (+)-GE3 [13] could exert substantial antitumor effects against BALB/c-nu/nu nude mice xeno grafted with currently incurable PSN1-human pancreatic carcinoma that a single 2 mg kg^−1^ dosage of (+)-GE3 was found to produce a 47 % reduction in tumor volume (11 days post-treatment), and none of the treated animals died as a result of receiving the drug [17].

Although the remarkable pharmacological activity and distinctive structure are intriguing, the ATM biosynthetic gene cluster has not been unearthed and the precise biosynthetic mechanism also remained obscure. Here we describe the characterization of the ATM biosynthetic gene cluster as well as the rational enhancement of its production. Genetic and biochemical experiments coupled with bioinformatics analysis demonstrated the essential role of the gene cluster involved in ATM biosynthesis, thereof leading to a proposed pathway for ATM biosynthesis. Further rational enhancement of ATM production has also been realized by tandem overexpression of the two pathway-specific regulatory genes *artB* and *artX*. The availability of the ATM biosynthetic gene cluster will lay a solid foundation for the mechanistic insight of ATM biosynthesis, and pave the way for the construction of high-producing industrial strains and for the rational generation of the designer ATM derivatives with enhanced/selective bioactivity via synthetic biology strategies.

## Results and discussion

### Identification and analysis of the aurantimycins gene cluster (*art*)

ATM shares a similar cyclohexadepsipeptide scaffold to polyoxypeptin, whose biosynthetic pathway has been previously identified from *Streptomyces* sp. MK498-98 F14 [12], implicating that some specific enzymes, in particular NRPSs, of both pathways should exhibit certain homology. Accordingly, we utilized the NRPSs (PlyF, PlyG and PlyH) from polyoxypeptin pathway as individual probes to conduct the Blastp analysis against the genome of *S. aurantiacus*, which directly resulted in the identification of a continuous region in *seq 14* (NZ_AOPZ01000014.1) involving homologs designated as ArtF (STRAU_0335, 55 % identity to PlyF), ArtG (STRAU_0336, 51 % identity to PlyG) and ArtH (STRAU_0337, 43 % identity to PlyG). Further looking through the context of the target region, other proteins (ArtE and ArtI) also show significant homologies to their counterparts (PlyE and PlyI) in polyoxypeptin pathway, all of these implicated that the target gene cluster is most likely involved in ATM biosynthesis.

**Fig. 2:**
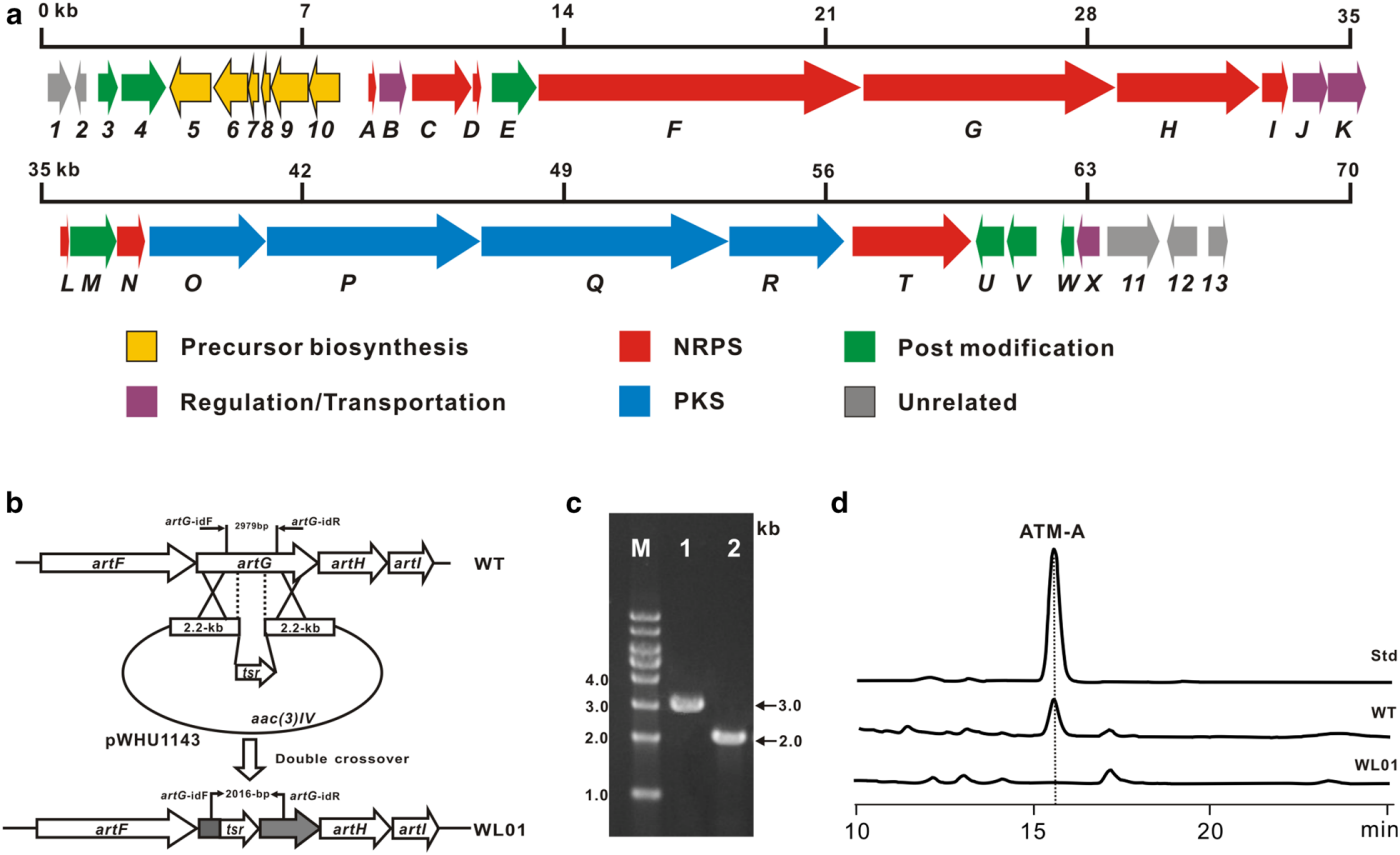
Organization and confirmation of the ATM biosynthetic gene cluster. **a** Organization of the ATM gene cluster. The proposed gene functions are listed in Table 1. **b** Schematic construction of the *ΔartG* mutant WL01. **c** Confirmation of the *ΔartG* mutant WL-01 by PCR. *1* wild type; *2* the *ΔartG* mutant WL01; M, 1 kb ladder. **d** HPLC analysis of the extract samples of *S. aurantiacus* JA 4570 wild type and the mutant WL01

To test our postulation’s validity, a*rtG* (encoding a NRPS enzyme) disruption plasmid pWHU1143 was constructed (Fig. 2b) and introduced into *S. aurantiacus* via conjugation. On the basis of the standard protocols, the mutant WL01 was screened and validated by PCR (Fig. 2c). After fermentation, the extract samples of WL01 and wild type strains were submitted for HPLC analysis. As expected, the sample of WL01 mutant was not capable of generating the characteristic ATM-A peak (Fig. 2d; Additional file 1: Figure S1), thus demonstrating that the target gene cluster is responsible for ATM biosynthesis.

Bioinformatic analysis revealed that* seq 14* (NZ_AOPZ01000014.1), comprising 36 deduced open reading frames (ORFs) (Table 1), probably houses the entire ATM biosynthetic gene cluster (Fig. 2a). Amongst of them, there are 4 putative NRPS genes (*artTFGH*) encoding 6 modules responsible for the template-synthesis of hexadepsipeptides, 4 PKS genes (*artOPQR*) for 4 modules that perfectly match the assembly line of C_14_ acyl chain based on the collinearity hypothesis [18], and it has 5 ORFs for 5 free-standing modules including one adenylation (A) domain (ArtC), two peptidyl carrier protein (PCP) domains (ArtD and ArtL), and two thioesterase (TE) domains (ArtI and ArtN) for participating in posttranslational modification or the formation of special monomers. In addition, it also contains one ORF for MbtH-like protein (ArtA), two ORFs for ATP-binding cassette (ABC) transporter (ArtJ and ArtK), and two ORFs for potential regulatory proteins (ArtB and ArtX).

**Table 1:** Deduced functions for the *orfs* in ATM biosynthetic gene cluster

Protein	Size^a^	Accession no.	Proposed function	Homolog. origin	Identity/similarity
Orf1	206	EFE72573	Sugar transporter	SSFG_07808, *Streptomyces ghanaensis* ATCC 14672	82/88
Orf2	91	EME58614	Phosphopantetheine attachment site domain-containing protein	H074_18583, *Amycolatopsis decaplanina* DSM 44594	35/65
Orf3	181	AKJ15912	NADH: riboflavin 5′-phosphate oxidoreductase	ABB07_39600, *Streptomyces incarnatus*	57/67
Orf4	393	EFE72565	Cytochrome P450 hydroxylase	SSFG_07800*, Streptomyces ghanaensis* ATCC 14672	76/88
Orf5	378	KTF47283	Zinc-binding dehydrogenase	APS67_01215, *Streptomyces *sp. AVP053U2	82/90
Orf6	312	EFE73053	3-Oxoacyl-[acyl-carrier-protein] synthase III	SSGG_00419, *Streptomyces roseosporus* NRRL 15998	71/82
Orf7	82	KMO67717	Phosphopantetheine attachment site domain-containing protein	MCHLDSM_06970, *Mycobacterium chlorophenolicum*	38/55
Orf8	77	XP_008613423	Dihydrolipoamide succinyltransferase	SDRG_09281, *Saprolegnia diclina* VS20	44/62
Orf9	326	KOX10942	2-Oxoisovalerate dehydrogenase	ADK66_07235, *Micromonospora *sp. NRRL B-16802	50/64
Orf10	279	KTF47288	3-Methyl-2-oxobutanoate dehydrogenase	APS67_01220, *Streptomyces *sp. AVP053U2	86/91
ArtA	71	KOG32626	MbtH-like protein	ADK37_26530, *Streptomyces resistomycificus*	87/94
ArtB	240	KUN90566	Putative regulator	AQJ84_39630, *Streptomyces resistomycificus*	77/86
ArtC	530	KUM93570	A	AQI88_26200, *Streptomyces cellostaticus*	84/90
ArtD	77	AKJ15824	PCP	ABB07_39115, *Streptomyces incarnatus*	84/90
ArtE	395	KUM93571	FAD-dependent oxidoreductase	AQI88_26210, *Streptomyces cellostaticus*	81/90
ArtF	2882	KUM93572	C-A-PCP-E-C-A-MT-PCP	AQI88_26215, *Streptomyces cellostaticus*	81/88
ArtG	2574	KOU40387	C-A-PCP-E-C-A-PCP	ADK54_22435, *Streptomyces *sp. WM6378	78/86
ArtH	1274	KJY47954	C-A-PCP-TE	VR46_00735, *Streptomyces *sp. NRRL S-444	78/87
ArtI	248	KUM93575	TE	AQI88_26230, *Streptomyces cellostaticus*	86/90
ArtJ	312	KOG32633	ABC transporter	ADK37_26575, *Streptomyces resistomycificus*	86/92
ArtK	253	KIF05972	ABC transporter permease	PL81_10120, *Streptomyces *sp. RSD-27	81/92
ArtL	73	KUM93581	PCP	AQI88_26270, *Streptomyces cellostaticus*	86/90
ArtM	416	KUM99154	Cytochrome	AQI95_40375, *Streptomyces yokosukanensis*	89/93
ArtN	245	KUM93583	TE	AQI88_26280, *Streptomyces cellostaticus*	79/87
ArtO	1034	KUM93584	KS-AT-ACP	AQI88_26285, *Streptomyces cellostaticus*	80/86
ArtP	1899	KUM93585	KS-AT-DH-KR-ACP	AQI88_26290, *Streptomyces cellostaticus*	71/79
ArtQ	2199	KUM93586	KS-AT-DH-ER-KR-ACP	AQI88_26295, *Streptomyces cellostaticus*	77/85
ArtR	1040	KJY47935	KS-AT-ACP	VR46_00775, *Streptomyces *sp. NRRL S-444	81/89
ArtT	1061	KUM93588	C-A-PCP	AQI88_26305, *Streptomyces cellostaticus*	72/81
ArtU	248	AGK78157	DUF1838	SFUL_3223, *Streptomyces fulvissimus* DSM 40593	89/93
ArtV	260	KUL64059	Peptidoglycan-binding protein	ADL30_00970, *Streptomyces *sp. NRRL S-1521	77/83
ArtW	116	CAJ88026	Conserved hypothetical protein	SAMR0316, *Streptomyces ambofaciens* ATCC 23877	78/82
ArtX	209	KOT40573	TetR family Transcriptional regulator	ADK41_12650, *Streptomyces caelestis*	90/93
Orf11	467	AJE84472	Integrin-like protein	SLNWT_4096, *Streptomyces albus*	82/88
Orf12	261	AGM04325	Putative ArsR family transcriptional regulator	AORI_1737, *Amycolatopsis orientalis* HCCB10007	45/60
Orf13	176	AKJ08646	Hypothetical protein ABB07_00890	ABB07_00890, *Streptomyces incarnatus*	49/62

Orf1(STRAU_0320)*…………*Orf12(STRAU_0357)

^a^Numbers are in amino acids

### ArtB and ArtX are the positive regulators during ATM biosynthesis

Within the *art* gene cluster, there are two putative regulatory genes *artB* and *artX* speculated to govern ATM production. Between them, ArtB shows significant homology to a large family of uncharacterized potential regulators (Additional file 1: Figure S2) and was conjectured to be a novel pathway-specific regulator class. Secondary structural analysis using SWISS-MODEL [19] indicated that it had a HTH motif, which was likely to bind to promoter region of the target gene to participate in regulating process. *artX,* located in the right end of the cluster, was deduced to encode a TetR-like protein, which displayed high identity to other TetR-family regulators (TFRs) (90 % identity to ADK58_14020 of *Streptomyces* sp. XY152). According to the analysis by online program ESPript 3.0 [20], ArtX might contain nine potential helixes (Additional file 1: Figure S4).

**Fig. 3:**
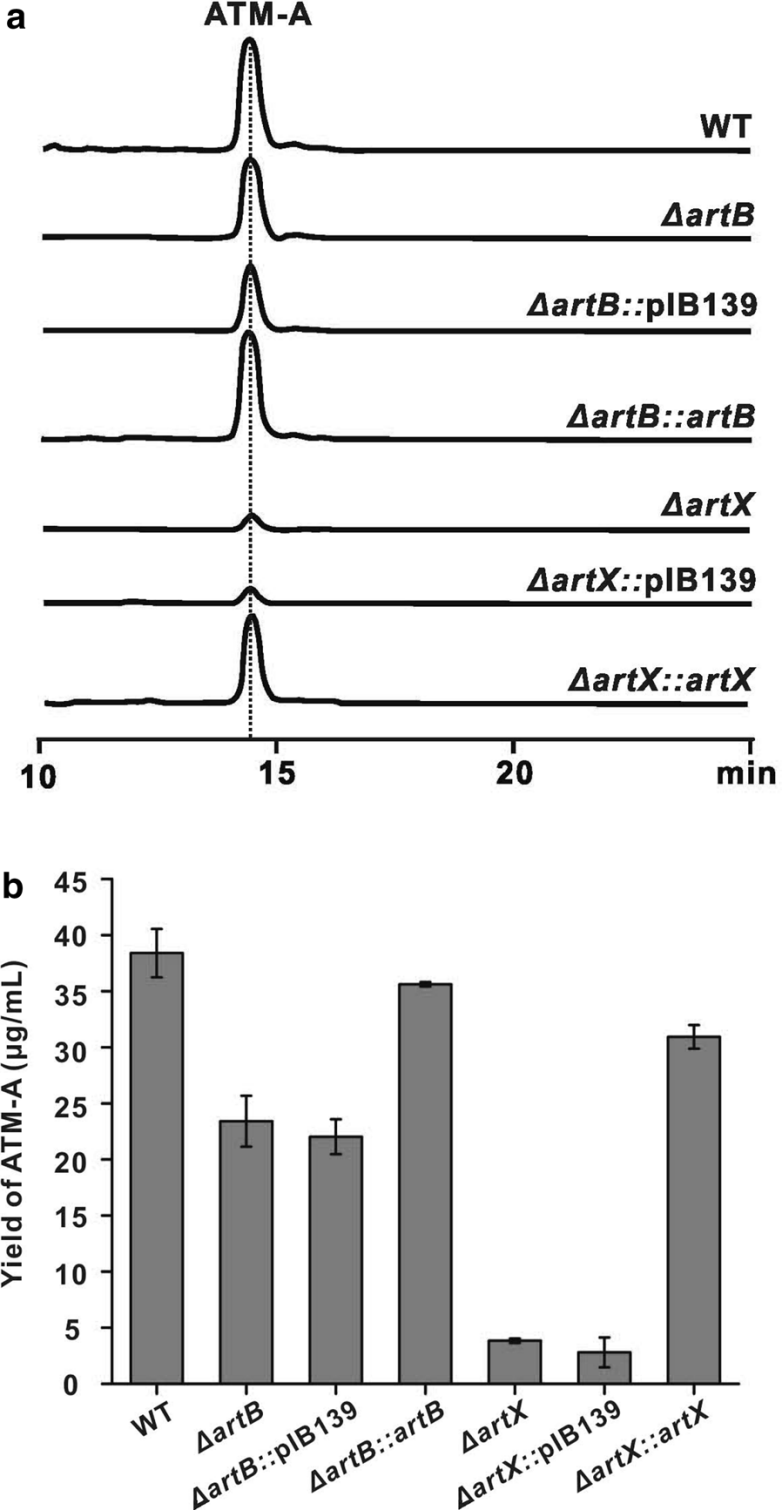
Genetic characterization of *artB* and *artX* as positive regulatory genes for ATM biosynthesis. **a** HPLC analysis of ATM-A production by related recombinants of *S. aurantiacus*. WT, wild type *S. aurantiacus* JA 4570; *ΔartB*, the *ΔartB* mutant WL02 of *S. aurantiacus*; *ΔartB*::pIB139, *ΔartB* mutant WL02 containing pIB139 as negative control; *ΔartB::artB, ΔartB *mutant WL02 containing pIB139/*artB* for complementation; *ΔartX*, the *ΔartX* mutant WL03 of *S. aurantiacus*; *ΔartX*::pIB139, *ΔartX* mutant WL03 containing pIB139 as negative control; *ΔartX::arX, ΔartX *mutant WL03 containing pIB139/*artX* for complementation; **b** Comparative analysis of ATM-A production produced by related recomniants of *S. aurantiacus*. All measurements were conducted in triplicate

To functionally characterize the two putative regulators, the disruption vectors pWHU1144 (Additional file 1: Figure S3A) and pWHU1145 (Additional file 1: Figure S5A) were individually constructed and conjugated into *S. aurantiacus*. According to the standard methods [21], the individual mutants of *artB* and *artX* were screened and further confirmed by PCR (Additional file 1: Figures S3B, S5B). The *artB and artX* mutants, correspondingly designated as WL02 and WL03, were inoculated for fermentation. Subsequently, the fermentation broth of the mutants and wild type strain were purified for HPLC analysis. The results showed that the ATM-A production by WL02 mutant decreased to about 45 %, and that by WL03 was significantly reduced to only 10 % (Fig. 3a, b; Additional file 1: Table S3). When the mutants were complemented by individual genes (cloned into pIB139), the ATM-A production for the both complemented strains were partly restored (Fig. 3a, b). All of these data demonstrated that both of ArtB and ArtX are the pivotal activators in ATM biosynthesis.

Actually, TetR-family regulators (TFRs) are widespread in Actinomycetes to serve as either activators or repressors during secondary metabolite biosynthesis, and certain species of actinomycetes have over 100 TFRs, including *S. coelicolor* (153 TFRs), *Streptomyces avermitilis* (115 TFRs), and *Streptomyces griseus* (104 TFRs) [22]. They consist of two domains: a conserved helix-turn-helix DNA binding domain (DBD) close to N-terminus, and a C-terminal bacterial transcriptional activation domain (BTAD) that displays broad sequence and structural variation and interacts with a wide variety of ligands [23]. In this respect, ArtX might act in a similar way to control ATM biosynthesis.

### Overexpression of *artB or/*and *artX* boosts ATM production

**Fig. 4:**
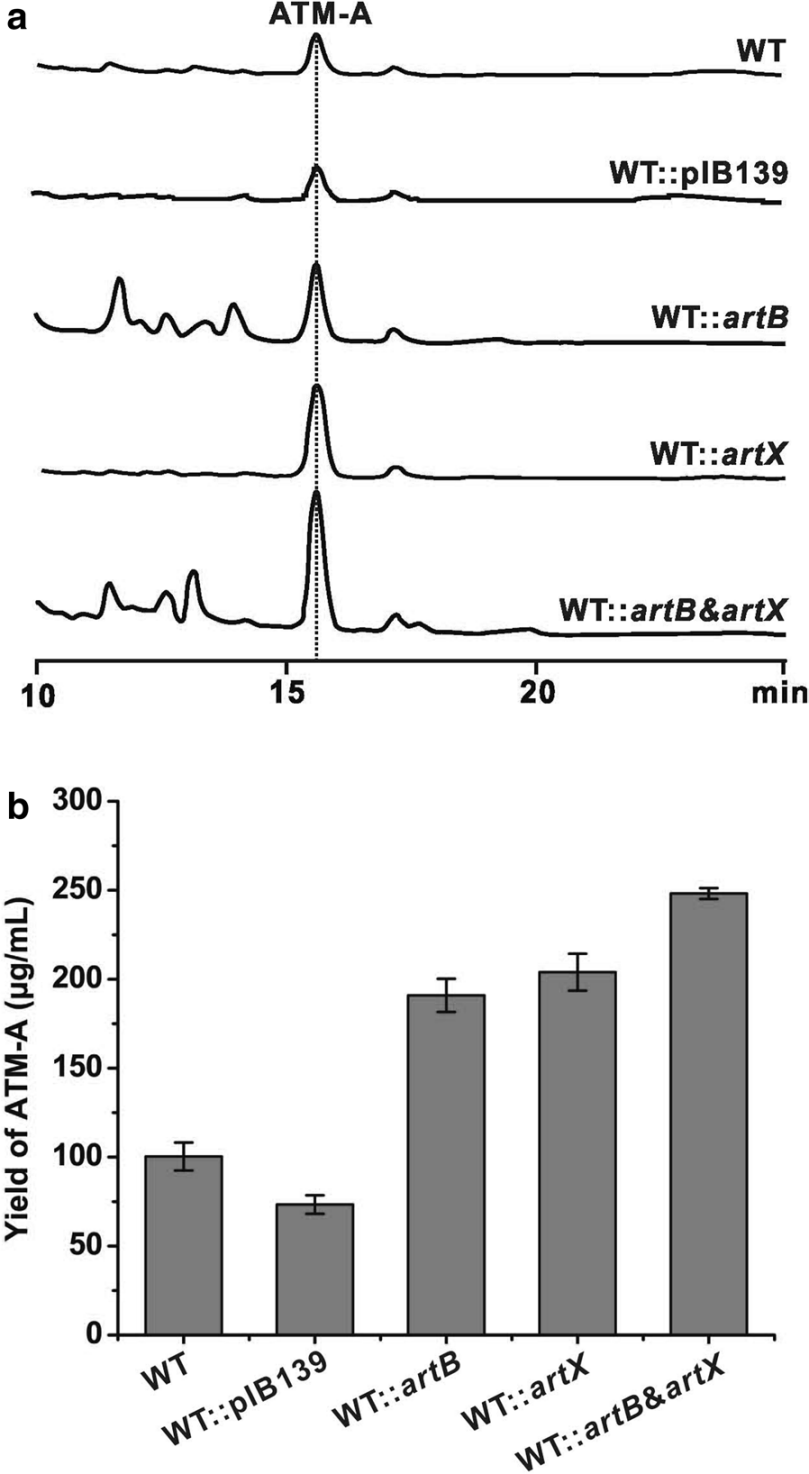
Individual overexpression *of artB, artX* as well as *artB & artX* in *S. aurantiacus* remarkably enhances ATM-A production. **a** HPLC analysis of the ATM-A production by relevant *S. aurantiacus* recombinants. **b** Comparative analysis of the ATM-A production by target strains. WT, *S. aurantiacus* wild type; WT::pIB139, *S. aurantiacus* containing pIB139 as negative control; WT*::artX*, *S. aurantiacus* containing pIB139/*artX*; WT*::artB*, *S. aurantiacus* containing pIB139/*artB;* WT*::artB* & *artX*, *S. aurantiacus* containing pIB139/*artB & artX.* All measurements were conducted in triplicate

Since both ArtB and ArtX are the positive regulators to control ATM biosynthesis, introduction of an extra copy of them is probably a feasible way to increase the antibiotic production. To test this speculation, *artB* and *artX,* therefore, were independently cloned into pIB139 under the control of PermE* and then the resultant plasmids were conjugated into the wild type strain of *S. aurantiacus*. After PCR confirmation (Additional file 1: Figure S6A, S6B), the conjugants (WT::*artB* and WT::*artX*) were inoculated for fermentation for 5 days. Subsequently, the extract samples of the fermentation broth were subjected for HPLC analysis. Conforming to the anticipation, introduction of an extra copy of *artB* or *artX* was capable of increasing the ATM-A production to 1.9- and 2.0-fold (Fig. 4a, b; Additional file 1: Table S4).

Rewarded by the production enhancement via *artB* or *artX* overexpression, we were promoted to ponder if we could construct higher yield strains by simultaneously manipulating the two activators. Then we tandemly overexpressed both *artB* and *artX* in wild type strain of *S. aurantiacus*. The validated recombinant strains (Additional file 1: Figure S6C) were inoculated for fermentation, and the extract sample of the recombinant WT::*artB* & *artX* were submitted for HPLC evaluation. The results indicated that ATM-A production by WT::*artB* & *artX* was increased to ca. 2.5-fold compared with that of the wild type strain (Fig. 4a, b). All these illustrated that simultaneous overexpression of two positive regulatory genes was a feasible and effective approach for the rational enhancement of ATM-A production, and might be extended for the overproduction of other industrially and clinically important antibiotics.

### Adenylation (A) domain activity assays define their independent roles for ATM biosynthesis

**Fig. 5:**
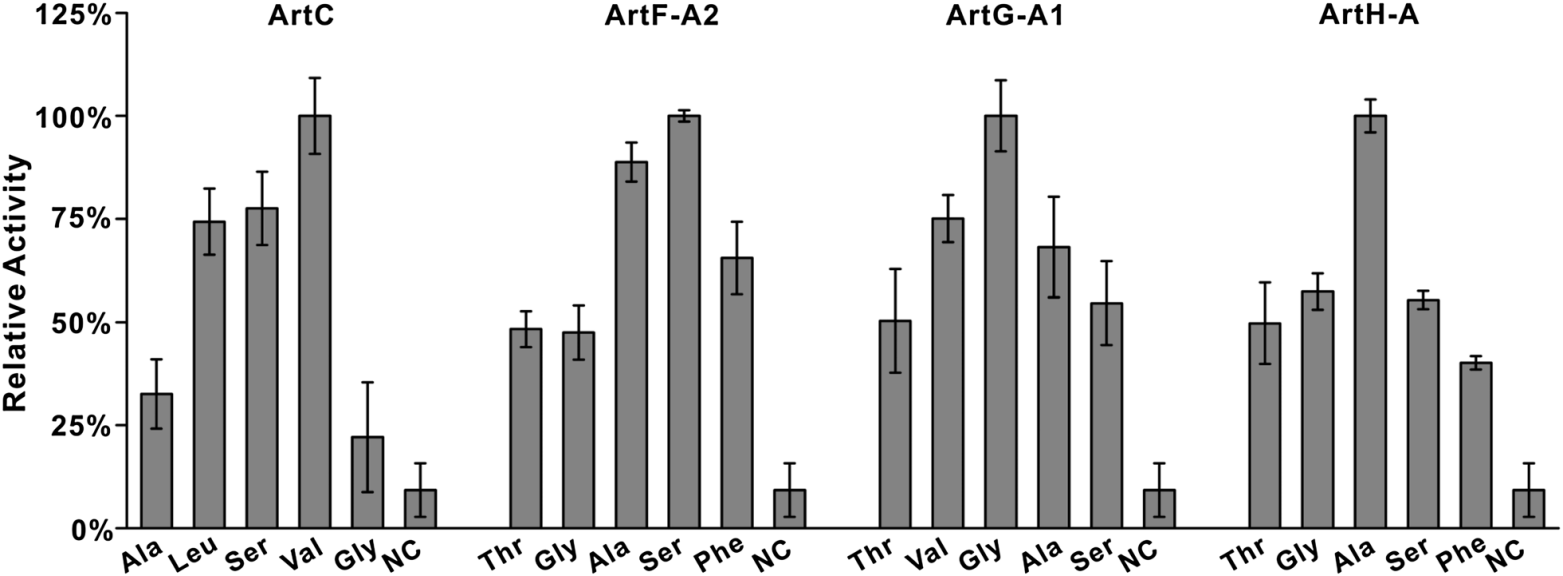
Substrate specificities of four adenylation domains ArtC, ArtF-A2, ArtG-A1, and ArtH-A in vitro. *NC,* no amino acid substrate. All amino acid substrates (4 mM) are L type, unless otherwise indicated. The background has been subtracted. *Error bars* represent standard deviations from three independently performed experiments

A domains function as the gatekeepers of NRPSs since they select, activate, and then install the carboxylic acid substrates onto the adjacent PCP domain in the biosynthesis of nonribosomal peptides. And analysis of primary sequence and key amino acid residues of substrate-binding pockets of these domains allows for the prediction of A domains preferences. Within the *art* gene cluster, the A domains have predicted substrate specificities in agreement with the cyclodepepsipeptides scaffold of ATM. To test the proposed specificities of four A domains of seven, ArtC, ArtF-A2, ArtG-A1, ArtH-A were individually overexpressed and purified from *E. coli* (Additional file 1: Figure S7), and further tested their substrate activities in vitro. The results indicated that the most favorable substrate for ArtF-A2 is L-Ser, while the preferred amino acids for ArtG-A1 and ArtH-A are Gly and L-Ala, respectively (Fig. 5). All of the data were fully consistent with the results of bioinformatic analysis.

More notably, we revealed that ArtC is a freestanding. A domain enzyme in the ATM biosynthetic pathway, implicating that such enzyme is most likely to harbor the function of trans-activating more than one amino acids. To validate such hypothesis, we test its activity in vitro with five selected substrates, and the results indicated the most preferred substrate for this enzyme is L-Val, and interestingly the other two amino acids L-Ser and L-Leu can also be recognized and activated by the enzyme. These combined data are completely matched with the A domain predication results and the assembly logic for ATM biosynthesis.

### Biosynthesis and assembly of the C_14_ acyl side chain

**Fig. 6:**
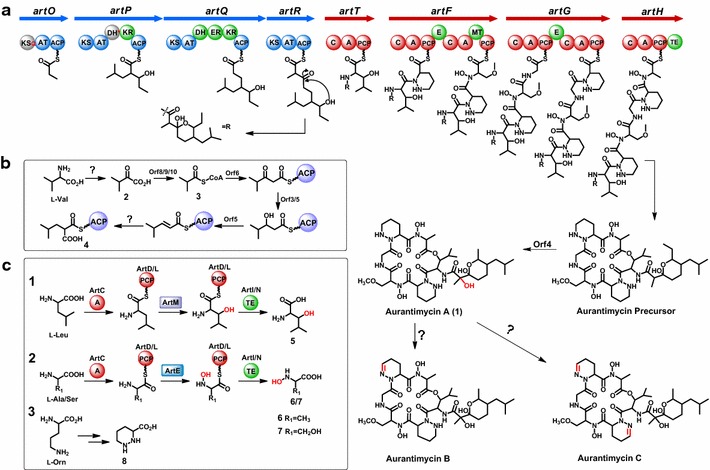
Proposed biosynthetic pathway for ATM. **a** The proposed model for ATM scaffold assembly line driven by the hybrid PKS/NRPS system. *KS* ketosynthase, *AT* acyltransferase, *ACP* acyl carrier protein, *DH* dehydratase, *KR* ketoreductase, *ER* enoylreductase, *A* adenylation domain, *PCP* peptidyl carrier protein, *C* condensation domain, *MT* methyltransferase domain, *E* epimerase domain, *TE* thioesterase domain. **b** The proposed pathway for the biosynthesis of 2-(2-methylpropyl)malonyl-ACP; **c** The proposed pathway for the biosynthesis of the nonproteinogenic amino acids building blocks

Within the *art* cluster, the organization of 4 type I PKS modules encoded by *artOPQR* coincides with the assembly line of the C_14_ acyl side chain of ATM via three steps of elongation from the propionate starter unit (Fig. 6a). Both ArtO and ArtR consist of ketosynthase (KS), acyltransferase (AT), and acyl carrier protein (ACP). However, the active site Cys (for *trans* thioesterification) of the ArtO-KS is substituted with Gln, a devitalizing phenomenon named “KS_Q_” that often occurs in the loading module of PKS system [24] (Additional file 1: Figure S8). Therefore, ArtO serves as a loading module for the formation of propionate starter unit by catalyzing decarboxylation of methylmalonyl group after tethering onto ACP (Fig. 6a). The conserved regions of AT domain including the active motif GHSQG [25] in both ArtO and ArtR, along with substrate specificity code YASH [26] indicate that both ATs are specific for methylmalonyl-CoA, consistent with the structure of the side chain of ATM (Fig. 6a).

ArtQ, consisting of an active DH domain, an ER domain, and an AT domain, contains the active site HAFH, so it was assumed to specifically recognize malonyl-CoA as the extender. In ArtP, except for three core domains KS, AT, and ACP, a dehydratase (DH) domain and a ketoreductase (KR) are also present. On the basis of collinearity of the domain architecture to the ATM structure, the DH domain here is believed to be nonfunctional, however, why this confusing phenomenon occurs in ATM assembly line remained unknown. In addition, the conserved motif of ArtP-AT for substrate selectivity is VPGH, neither the serine residue in YASH for methylmalonyl-CoA nor phenylalanine residue in HAFH for malonyl-CoA. These changes may broaden the substrate binding pocket and enhance its hydrophobicity, supporting that ArtP likely recognizes 2-(2-methylpropyl) malonyl-CoA as unusual extender unit (Fig. 6b).

### Assembly of the cyclodepepsipeptide by NRPSs

After the C_14_ acyl side chain is synthesized by 4 modular PKSs, it is transferred to 3-hydroxyleucine with formation of an amide bond catalyzed by ArtT, thus initiating the assembly of the peptide core architecture. The *art* gene cluster encompasses four genes *artTFGH* coding for a multimodular NRPS that comprises 6 extension modules, in agreement with 6 condensation steps required for the biosynthesis of the ATM cyclodepsipeptide scaffold (Fig. 6a).

Both ArtF and ArtG consist of two modules that are correspondingly composed of seven domains (C-A1-PCP-E-C-A2-PCP) (Fig. 6a). The ArtF-A1 and ArtG-A2 are proposed to recognize and activate L-piperazic acid that was speculated to be derived from L-ornithine [27]. This assumption can be supported by the findings that ArtF-A1 shares 52–66 % identity with ArtG-A2, HmtL-A1, and KtzH-A1 (Additional file 1: Figure S10); more convincingly, the conserved substrate specificity-conferring amino acids (DVFSVASYAK for ArtF-A1, DVFTVAAYAK for ArtG-A2) are highly analogous to those of KtzH-A1 (DVFSVGPYAK) and HmtL-A1 (DVFSVAAYAK), which were reported to recognize and activate piperazic acid in previous studies [28, 29]. All of these suggested that ArtF-A1 and ArtG-A2 are probably to specifically recognize and transfer piperazic acid to the corresponding PCP domain.

In the middle of the modules of ArtF and ArtG, an active epimerase (E) domain is present, indicating that the amino acids activated by ArtF-A1 and ArtG-A1 should be converted in D-configuration. Confusingly, the very amino acid activated by ArtG-A1 is glycine in actual, which means the E domain, either functional or nonfunctional, could not affect the configuration of this building block, but why an E domain present in the ArtG enzyme remains mysterious. Moreover, this may be also the reason that two piperazic acids have opposite configurations in ATM.

There is an *O*-methylation in the L-Ser residue of ATM which is usually catalyzed by an *O*-methyltranferase. Scanning through the whole *art* gene cluster, while we did not identify the target enzyme likely involved in such methylation, therefore we reinvestigated the domain organization of ArtF, rendering the discovery of a methyltransferase (MT) domain which was proposed to be responsible for the *O*-methylation during the assembly of cyclohexadepsipeptide scaffold.

As for ArtH, it contains four domains (C-A-PCP-TE) as predicated by in silico analysis, the terminal TE domain present in ArtH strongly demonstrated that this enzyme was the last module of ATM NRPS-template system and responsible for the release and cyclization of the peptide chain via ester bond formation.

### Biosynthesis of the unusual extender unit 2

Based on the structural analysis of ATM architecture, a 2-(2-methylpropyl) malonyl extender unit (2) is probably incorporated into the assembly line of C_14_ moiety. Investigation of the *art* gene cluster, *orf2*-*orf10* (Fig. 2a), surprisingly, are completely matched to those in polyoxypeptin gene cluster, in either organization or homology. Thus, we concluded that those genes were responsible for the unusual moiety’s biosynthesis (Fig. 6b).

For the initiating the biosynthesis of the unusual extender unit, L-Val was deduced as the starter substrate which was followed by a transamination to form compound 2, whereas we could not assign an obvious protein from the ATM pathway for such catalyzed step. Similar reaction has been reported for formation of the 2-(2-methylbutyl) malonyl unit of polyoxypeptins derived from isoleucine [27]. After that, the chain of 3 was believed to elongate by condensation with α-keto acid catalyzed by Orf6, a protein shares 71 % similarity and 82 % identity with SSGG_00419 that was a 3-oxoacyl-[acyl-carrier-protein] synthase III from *Streptomyces roseosporus* NRRL 15998, with the assistance of Orf7 as speculated as a phosphopantetheine attachment site containing protein. Within the *art* gene cluster, *orf9* codes for a 2-oxoisovalerate dehydrogenase homolog to ADK66_07235 of *Micromonospora* sp. NRRL B-16802 (identity/similarity, 50/64 %), and *orf10* for a 3-methyl-2-oxobutanoate dehydrogenase to APS67_01220 from *Streptomyces* sp. AVP053U2 (identity/similarity, 89/91 %), and they might mediate the previous acylation reaction. Then the gene *orf5* encodes a zinc-binding dehydrogenase, suggesting it might participate in the subsequent intermediate’s ketoreduction. Finally, the generation of 2-(2-methylpropyl)malonyl unit (4) would involve a crotonyl-like reductive carboxylation mediated by some endoreductases homologs, although no such gene have been found in the *art* gene cluster (Fig. 6b).

### Biosynthesis of the nonproteinogenic amino acid building blocks

Except for the modular NRPSs, there are five free-standing genes present in the *art* gene cluster deduced to individually encoding an A domain (ArtC), two PCP domains (ArtD and L) and two TE domains (ArtI and N). Since the discrete NRPS domains found in many NRPS assembly lines are often responsible for the formation of nonproteinogenic building blocks [18]. These enzymes were assumed to catalyze the generation of these monomers from natural amino acids, and ArtC was characterized to activate multiple amino acids as Ala, Ser, and Val, and tether them to the corresponding PCPs (ArtD or ArtL). After *N*-hydroxylation of serine and alanine (6/7) as well as β-hydroxylation of leucine (8), the matured building blocks are proposed to be released by discrete TEs, either ArtI or ArtN (Fig. 6c), and then activated by the subsequent corresponding modular A domains for the assembly of the ATM scaffold.

Piperazic acid is an attractive building block of many complex secondary metabolites such as antrimycin [30], chloptosin [31], himastatin [32], luzopeptin [33], quinoxapeptin [34], lydiamycin [35], piperazimycin [36] and sanglifehrin [37], while the detailed piperazic acid biosynthetic mechanism still remained poorly understood yet. Previously, Walsh and coworkers demonstrated that KtzI, a homolog of lysine/ornithine *N*-hydroxylases catalyzed the conversion of ornithine into *N*-hydroxy-ornithine [38, 39], but how this intermediate was further utilized to form piperazic acid remained totally unknown and confused the biochemists for over half a century (Fig. 6c).

### The tailoring steps for ATM biosynthesis

After maturation, the ATM precursor was released by TE domain of the NRPS assembly line, whereas it needs further tailoring steps for the fulfillment of the ATM biosynthesis. Within the *art* gene cluster, a candidate gene *orf4* coding for a cytochrome P450 hydroxylase was tentatively assigned to be responsible for the tailoring hydroxylation to synthesize the matured ATM-A. Interestingly, three ATM components (ATM-A, B and C) differed in the extent of unsaturation of two opposite piperazic acid, but how such enzymatic reaction (oxidation/reduction) occurs remained enigmatic.

## Conclusions

Azinothricin family natural products have spurred an increasing interest in recent years due to their unusual structural features and diverse bioactivities. However, the knowledge on the biosynthetic gene cluster and pathway of this family of antibiotics is limited. In combination of genetic, biochemical, and bioinformatics analysis, we report the identification and characterization of the ATM biosynthetic gene cluster, and delineate the proposed assembly line for ATM biosynthesis. More importantly, we illustrate the positive roles of ArtB and ArtX in ATM biosynthesis, and further realize the rational enhancement of ATM production via tandem overexpression of *artB* and *artX*. We anticipate that manipulation of the ATM pathway would be of great potential to generate novel ATM-based antibiotics with enhanced activity and modified selectivity.

## Methods

### Bacterial strains, plasmids, and culture conditions

Bacterial strains and plasmids used in this study are listed in Table S1 and the relevant PCR primers in Table S2. *Escherichia coli* strains including DH10B, ET12567/pUZ8002, and Rossetta (DE3) were cultivated at 37 °C in Luria–Bertani (LB) liquid medium or on LB agar. *S. aurantiacus* JA 4570 and its derivatives were cultured at 28 °C in TSBY (yeast extract 5 g/L, tryptic soy broth 30 g/L, sucrose 103 g/L) for DNA extraction and on Mannitol-Soybean (MS) agar for sporulation and genetic manipulation. For fermentation, mycelia of the strain JA 4570 and its derivatives were inoculated in fermentation medium (soybean powder 20 g/L, glucose 10 g/L, NaCl 5 g/L, CaCO_3_ 3 g/L, pH7.2) and cultivated at 28 °C for 5 days. When necessary, the medium was supplemented with apramycin 30 μg/mL, chloramphenicol 25 μg/mL, kanamycin 50 μg/mL, thiostrepton 12.5 μg/mL, or ampicillin 100 μg/mL.

### Construction of the target mutants

For the construction of the target mutants, the target gene disruption plasmids were transformed into *E. coli* ET12567/pUZ8002 cells, and the conjugation between *E. coli* ET12567/pUZ8002 and *S. aurantiacus* was performed on MS medium. The exconjugants confirmed by PCR were further inoculated in a new TSBY without antibiotic added for 8 days. Subsequently, all the mutant strains in this study were generated and screened according to the standard method [21]. The randomly selected thiostrepton-resistant and apramycin-sensitive candidates were confirmed by PCR with corresponding primers listed in Additional file 1: Table S2.

### Complementation of the *artB* or *artX* mutants and construction of *artB* or/and *artX* overexpression strains

All the overexpression strains are generated by lysogenic recombination according to the standard method [21]. For the complementation of *artB* or *artX* mutants, the target gene *artB* or *artX* was cloned into the vector pIB139 under control of PermE*. After confirmation, the recombinant plasmid was transformed into *E. coli* ET12567/pUZ8002 cells. Then *artB* (*artX*) cloned in pIB139 was conjugated into *artB* (*artX*) mutant or conjugated into wild type strain of *S. aurantiacus* for ATM overproduction. For the tandem overexpression of *artB* and *artX*, pWHU1149 containing a*rtB* and *artX* was conjugated into *S. aurantiacus* for ATM overproduction. The exconjugants would appear three days later and the exconjugants confirmed by PCR with corresponding primers were cultivated for further fermentation.

### HPLC analysis of ATM production

After fermentation, the culture broth was centrifuged and the mycelium pellet was extracted by 5 volume of methanol overnight. The supernatant of methanol phase was concentrated to 25 mL by rotary evaporator under the reduced pressure, then 25 mL H_2_O and 50 mL n-hexane were added for the first extraction. Subsequently, the product was extracted by 50 mL trichloromethane in the second extraction. Finally, trichloromethane was swapped-off by the rotary evaporator and then the residue was dissolved in methanol (500 μL) for HPLC analysis. The HPLC analysis was conducted on a Shimadzu LC-20A equipped with a SB-18 column (Shimadzu, 3.5 µm, 4.6 × 250 mm), the conditions are as follows: 80 % B (isocratic elution, solvent B is acetonitrile) at the flow rate of 0.5 mL/min with reverse-phase column at 28 °C.

### Adenylation domain cloning, expression, purification, and assays

Structural genes containing the corresponding adenylation domains ArtF-A2, ArtG-A1 and ArtH-A, and independent A domain protein ArtC were amplified from *S. aurantiacus* genome DNA via KOD Fx high fidelity PCR polymerase (TOYOBO) with individual pair of primers listed in Additional file 1: Table S2. The Purified PCR products were first cloned into pEASY Blunt vector, after confirmation by DNA sequencing, the related *Nde*I-*Eco*RI engineered structural genes were individually cloned into pET-28a (+) following standard protocols. The resulting expression constructs were subsequently transformed into *E. coli* Rosetta (DE3) cells, and the resulting transformants were grown in LBBS medium (Betaine 0.309 g/L, Sorbitol 185.9 g/L, yeast extract 5 g/L, tryptone 10 g/L, NaCl 5 g/L) supplemented with antibiotics kanamycin 50 μg/mL and chloromycin 25 μg/mL. Expression and purification for all His6-tagged proteins followed the same general procedures and is described as follows: in 1 L of liquid LBBS culture, the cells were grown at 37 °C until the OD_600_ of 0.6–0.8. The cells were cooled on ice for 10 min, and then induced with 0.12 mM IPTG for 72 h at 15 °C at 220 r. After that, the cells were harvested by centrifugation (5000 rpm, 10 min, 4 °C), resuspended in 30 ml lysis buffer (25 mM Tris, pH 8.0, 150 mM NaCl, 5 mM imidazole) and lysed by homogenization on ice. Cellular debris was removed by centrifugation (10,000 r/min, 40 min, 4 °C). The soluble proteins were incubated with 2 ml of Ni–NTA agarose resin (Qiagen), nutated in the ice overnight, and loaded onto a gravity flow column. The proteins were washed with washing buffer (25 mM Tris, pH 7.8, 150 mM NaCl) and eluted with elution solution (25 mM Tris, pH 7.8, 150 mM NaCl, and 200 mM imidazole). Purified proteins were concentrated and buffer exchanged into protein stock buffer (25 mM Tris, pH 8.0, 150 mM NaCl, and 10 % glycerol) using Amicon Ultra filters. Finally, the proteins were flash-frozen in liquid nitrogen and stored at −80 °C for further use.

ATP-PPi exchange assays were conducted by Enzcheck Pyrophosphate Assay Kit (E-6645) purchased from Thermo Fisher Scientific. Reaction was initiated by adding purified protein to a final concentration of 2 μΜ. After incubating at 22 °C for 45 min, the mixtures were transferred to 96-well plates and measured at 360 nm by Infinite M2000 Pro (TECAN).

### In silico analysis of the ATM gene cluster

The genome contig sequence was obtained from National Center for Biotechnology Information (NCBI) with accession number NZ_AOP01000014.1, and the deduced open reading frames (ORFs) were analyzed with the FramePlot 4.0beta online program (http://nocardia.nih.go.jp/fp4/) [40]. The function of putative proteins were deduced via homology-based analysis using the basic local alignment search tool (BLAST) program (http://www.ncbi.nlm.nih.gov/blast/) [41]. The NRPS-PKS architecture was analyzed by PKS/NRPS analysis program (http://nrps.igs.umaryland.edu/) online [42]. The multiple sequence alignment and analysis were performed by ClustalW on-line program (http://www.genome.jp/tools/clustalw/) [43]. The secondary structure was predicted by ESPript 3.0 (http://espript.ibcp.fr/ESPript/ESPript/) [20] and Swiss-Model (http://swissmodel.expasy.org/) [19].

## Additional file

10.1186/s12934-016-0559-7 Strains, plasmids, and cosmid used in this study. **Table S2.** PCR primers used in this study. **Table S3.** ATM-A Yields of related strains after culturing for 5 days in shake-flask for regulatory genes inactivation. **Table S4.** ATM-A Yields of related strains after culturing for 5 days in shake-flask for regulatory genes overexpression. **Figure S1.** HPLC analysis of aurantimycin A. **Figure S2.** Alignment of ArtB and other regulators from *Streptomyces*. **Figure S3.** Construction of WL-02 mutant. **Figure S4.** Alignment of ArtX with other TetR regulators. **Figure S5.** Construction of WL-03 mutants. **Figure S6.** Identification of target gene overexpression strains. **Figure S7.** SDS-PAGE analysis of four purified A domains. **Figure S8.** Alignment of four KS domains in PKSs of the art cluster. **Figure S9.** Alignment of A domains activating piperazic acid.

## Abbreviations

ATM: aurantimycin
ORF: open reading frame
NRPS: nonribosomal peptide synthetases
A domain: adenylation domain
PCP: peptidyl carrier protein
C domain: condensation domain
TE: thioesterase
E domain: epimerase domain
MT: methylase
KS: ketosyntheatase
AT: acyltransferase
ACP: acyl carrier protein
DH: dehydratase
ER: enoylreductase
KR: ketoreductase
TFR: TetR-family regulators
