# Supplementary material for: Characterization of the aurantimycin biosynthetic gene cluster and enhancing its production by manipulating two pathway-specific activators in Streptomyces aurantiacus JA 4570
Source: Microb Cell Fact. 2016 Sep 21;15:160. doi: 10.1186/s12934-016-0559-7 (PMC5031334; doi:10.1186/s12934-016-0559-7)
Supplement: Supplementary file 1 — 10.1186/s12934-016-0559-7 Strains, plasmids, and cosmid used in this study. Table S2. PCR primers used in this study. Table S3. ATM-A Yields of related strains after culturing for 5 days in shake-flask for regulatory genes inactivation. Table S4. ATM-A Yields of related strains after culturing for 5 days in shake-flask for regulatory genes overexpression. Figure S1. HPLC analysis of aurantimycin A. Figure S2. Alignment of ArtB and other regulators from Streptomyces. Figure S3. Construction of WL-02 mutant. Figure S4. Alignment of ArtX with other TetR regulators. Figure S5. Construction of WL-03 mutants. Figure S6. Identification of target gene overexpression strains. Figure S7. SDS-PAGE analysis of four purified A domains. Figure S8. Alignment of four KS domains in PKSs of the art cluster. Figure S9. Alignment of A domains activating piperazic acid. [file 12934_2016_559_MOESM1_ESM.pdf]

Supplemental Data

Characterization of the Aurantimycin Biosynthetic Gene Cluster and Enhancing Its Production by Manipulating Two Pathway-specific Activators in *Streptomyces aurantiacus* JA 4570

Houyuan Zhao,[1] Liang Wang,[1] Dan Wan,[1] Jianzhao Qi,[1] Rong Gong,[1] Zixin Deng,[1,2] Wenqing Chen[1*]

[1]Key Laboratory of Combinatorial Biosynthesis and Drug Discovery, Ministry of Education, and School of Pharmaceutical Sciences, Wuhan University, Wuhan 430071, China

[2]State Key Laboratory of Microbial Metabolism, and School of Life Sciences & Biotechnology, Shanghai Jiao Tong University, Shanghai 200030, China

[*]Address correspondence to: **Wenqing Chen,** Key Laboratory of Combinatorial Biosynthesis and Drug Discovery, Ministry of Education, and School of Pharmaceutical Sciences, Wuhan University, Wuhan 430071, China. **Tel**: +86-27-68756713, **E-mail**: wqchen@whu.edu.cn.

Contents of Supplemental Data

1. Supplemental Tables……………………………………………………………………………………3

Table S1: Strains, plasmids, and cosmid used in this study………………………………………3

Table S2: PCR primers used in this study……………………………………………………………5

Table S3: ATM-A Yields of related strains after culturing for 5 days in shake-flask for regulatory genes inactivation……………………………………………………………6

Table S4: ATM-A Yields of related strains after culturing for 5 days in shake-flask for regulatory genes overexpression………………………………………………………6

2. Supplemental Methods……………………………………………………………………………7

2.1 Construction of target gene disruption plasmids……………………………………………7

2.2 Generation of *artB*, *artX*, and *artB* & *artX* overexpression vectors……………………7

3. Supplemental Figures…………………………………………………………………………….8

Figure S1: HPLC analysis of aurantimycin A …………………………………………………8

Figure S2: Alignment of ArtB and other regulators from *Streptomyces*…………………9

Figure S3: Construction of WL-02 mutant………………………………………………………10

Figure S4: Alignment of ArtX with other TetR regulators……………………………………11

Figure S5: Construction of WL-03 mutants………………………………………………………12

Figure S6: Identification of target gene overexpression strains……………………………13

Figure S7: SDS-PAGE analysis of four purified A domains……………………………………14

Figure S8: Alignment of four KS domains in PKSs of the art cluster………………………15

Figure S9: Alignment of A domains activating piperazic acid…………………………………16

4. Supplemental References………………………………………………………………………17

1. Supplemental Tables

Table S1 Strains, plasmids, and cosmid used in this study

Strain, plasmids	Relevant genotype or description	Source/Reference
Streptomyces		
S. aurantiacus		
JA 4570	Wide-type strain producing aurantimycins	DMSZ [1]
WL01	*artG* mutant with insertion of *tsr*	This study
WL02	*artB* mutant with insertion of *tsr*	This study
WL03	*artX* mutant with insertion of *tsr*	This study
WT*::artB*	*artB* overexpression strain of *S. aurantiacus* JA4570	This study
WT*::artX*	*artX* overexpression strain of *S. aurantiacus* JA4570	This study
WT*::artB & artX*	A JA4570 derivative with *artX* and *artX* tandemly overexpressed	This study
ΔartB::artB	*artB* retro-complementation strains from WL02	This Study
*ΔartB::*pIB139	WL02 with introduction of vector pIB139	This Study
ΔartX::artX	*artX* retro-complementation strains from WL03	This Study
*ΔartX::*pIB139	WL03 with introduction of vector pIB139	This Study
E. coli		
DH10B	F^- *mcr*A Δ(*mrr-hsd*RMS-*mcr*BC) φ80d *lac*ZΔM15 Δ*lac*X74 *deo*R *rec*A1*end*A1*ara*Δ139 D(*ara*, *leu*)7697 *gal*U *gal*K λ^- *rps*L *nup*G	GIBCO BRL
ET12567(pUZ8002)	*dam dcm hsd*S pUZ8002	[2]
Rosetta(DE3)	F^- *ompT* $hsdS_B(r_B^- m_B^-)$ *gal dcm* (DE3) $pRARE^2$ (Cam^R)	Novagen
**Plasmids**		
pOJ446	*aa(3)IV,* SCP2, rep^{puc}, attΦC31, oriT	[3]
pIB139	*int, attp, aac(3)IV, PermE**	[4]
pJTU968	pRSET-B derivative, *bla*，*PermE **	[5]
pWHU1143-L	pOJ446 derivative carrying XbaI-BamHI engineered PCR fragment containing left arm of *artG* disruption vector	This study
pWHU1143-R	pWHU1143-L derivative carrying BamHI-EcoRI engineered PCR fragment containing right arm of *artG* disruption vector	This study
pWHU1143	pWHU1143-R derivative bearing BamHI engineered *tsr* fragment from pJTU2180 for *artG* mutation	This study
pWHU1144-L	pOJ446 derivative carrying XbaI-BamHI engineered PCR fragment containing left arm of *artB* disruption vector	This study
pWHU1144-R	pWHU1144-L derivative carrying BamHI-EcoRI	This study

	engineered PCR fragment containing right arm of *artB* disruption vector	
pWHU1144-*tsr*	pWHU1144-R derivative bearing BamHI engineered *tsr* fragment from pJTU2180 for *artB* mutation	This study
pWHU1145-L	pOJ446 derivative carrying XbaI-BamHI engineered PCR fragment containing left arm of *artX* disruption vector	This study
pWHU1145-R	pWHU1145-L derivative carrying BamHI-EcoRI engineered PCR fragment containing right arm of *artX* disruption vector	This study
pWHU1145-*tsr*	pWHU1145-R derivative bearing BamHI engineered *tsr* fragment from pJTU2180 for *artX* mutation	This study
pWHU1146	pIB139 derivative carrying NdeI-EcoRI digested PCR fragment containing *artB* from JA4570 genome for its overexpression	This study
pWHU1147	pIB139 derivative carrying NdeI-EcoRI digested PCR fragment containing *artX* from JA4570 genome for its overexpression	This study
pWHU1148	pJTU968 derivative bearing NdeI-EcoRI digested fragment containing *artX* from pWHU1147.	This study
pWHU1149	WHU1146 derivative carrying MfeI-EcoRI engineered fragment containing *PermE** & *artX* from WHU1148 for *artB & artX* double overexpression	This study
pEASY-Blunt	*lacZα*, pUC *ori*, *f1 ori,* Amp^r,	TransGen Biotech
pEASY-Blunt-*artC*	pEASY-Blunt derivative carrying *artC* fragments amplified by PCR	This study
pEASY-Blunt-*artF*-A2	pEASY-Blunt derivative carrying *artF*-A2 fragments amplified by PCR	This study
pEASY-Blunt-*artG*-A1	pEASY-Blunt derivative carrying *artG*-A1 fragments amplified by PCR	This study
pEASY-Blunt-*artH*-A	pEASY-Blunt derivative carrying *artH*-A fragments amplified by PCR	This study
pET28a(+)	*Kan, lacI,* BR322, *ori*	Novagene
pET28a-*artC*	pET28a(+) derivative carrying *artC* NdeI-EcoRI engineered fragments from pEASY-Blunt-*artC*	This study
pET28a-*artF*-A2	pET28a(+) derivative carrying *artF*-A1 NdeI-EcoRI engineered fragments from pEASY-Blunt-*artF*-A2	This study
pET28a-*artG*-A1	pET28a(+) derivative carrying *artG*-A1 NdeI-EcoRI engineered fragments from pEASY-Blunt-*artG*-A1	This study
pET28a-*artH*	pET28a(+) derivative carrying *artH* NdeI-EcoRI engineered fragments from pEASY-Blunt-*artH*	This study

**ori*T, origin of transfer of plasmid RK2; *tsr*, thiostrepton resistance gene; *aac(3)IV*, apramycin resistance gene; DMSZ: Deutsche Sammlung von Mikroorganismen und Zellkulturen.

Table S2. PCR primers used in this study

Primers	Sequence (5′ - 3′)
artG-LarmF	GCTCTAGAGAAGGACGCACCGACGAA
artG-LarmR	CGGGATCCGCGACGACCTGGATGGAA
artG-RarmF	CGGGATCCACCTGCTGCTGCTCTACGC
artG-RarmR	GGAATTCGCTGTTGCCCGCCTGTT
artG-idF	GACAGCATCCGTTCCATCC
artG-idR	CACCGAGTTCAACGACACG
artB-LarmF	GGGAACAGGACGAGAAGACGG
artB-LarmR	CCACGAAGGACAGCACCACAT
artB-RarmF	GGGTGACCCAGGTGCTCGAC
artB-RarmR	TCGGCAATCATCACAGTCCT
artB-idF	GAGCAGGTTGACCGAAGTGAA
artB-idR	CGACGAGCATGTCGATGGAGT
artB-expF	AGGACCGCGAGCAGTTACCG
artB-expR	CTAGGCGGTGCGCCGCTC
artX-LarmF	GCTCTAGACGTTGGAGTCCCAGTTGTTG
artX-LarmR	GAAGATCTCTTCTTCGGGCTGCTCTTC
artX-RarmF	GAAGATCTGCCTGCTTCTCGTTCTCCTC
artX-RarmR	GGAATTCCTCCGACCAACGCCTTCT
artX-idF	TGAAGAGCAGCCCGAAGA
artX-idR	CAGCACCTGAGGAGAACGAG
artX-expF	ATGACAGCACCTGAGGAGAACG
artX-expR	TCACCTGGCGCTCCCCGC
pIB139F	CGATGCTGTTGTGGGC
pIB139R	TGGCGATAAGTCGTGTCT
*tsr*F	TTGGACACCATCGCAAATC
*tsr*R	AAACCGAGGCGGAAGACG

ArtC-pro-expF	CGCCATATGGTCCACCAGCAGTTC
ArtC-pro-expR	GGAATTCACTATGCCGTGGCCGTGGT
ArtF-pro-expF	CGCCATATGCTGGAACAGCTGATCGAG
ArtF-pro-expR	GGAATTCAGCCGCGGGCGTACTCGCC
ArtG-pro-expF	CGCCATATGGCCGTCGCCGAACCGGGC
ArtG-pro-expR	GGAATTCAGTTCAGTTTCCCGTTCGG
ArtH-pro-expF	CGCCATATGGTCGTCGCCGACCCCGAC
ArtH-pro-expR	GGAATTCACGCGGGCAGCGCCCGCTG

F stands the forward primers, and R indicates the reverse primer.

Table S3 Yields of ATM-A for related strains after culturing for 5 days in shake-flask for regulatory genes inactivation

Strains	WT	*ΔartB*	*ΔartB::* pIB139	*ΔartB::artB*	*ΔartX*	*ΔartX::*pIB139	*ΔartX::artX*
ATM-A (μg/ml)	38.4 ± 1.8	23.4 ± 2.3	22.0 ± 1.6	35.6 ± 0.2	3.8 ± 0.2	2.8 ± 0.4	30.9 ± 1.1

Table S4 Yields of ATM-A for related strains after culturing for 5 days in shake-flask for regulatory genes overexpression

Strains	WT	WT::pIB139	WT*::artB*	WT*::artB*	WT*::artB*
ATM-A (μg/ml)	100.3 ± 1.8	73.4 ± 5.2	190.9 ± 9.4	203.9 ± 10.4	248.1 ± 3.0

2. Supplemental Methods

2.1 Construction of target gene disruption plasmids

For the construction of pWHU1143, the 2.2-kb homologous L- and R-arms for *artG* inactivation were amplified with primers *artG*-Larm-F/R and *artG*-Rarm-F/R. After treated by XbaI/BamHI, the left arm was cloned into the corresponding sites of pOJ446 to generate pWHU1143L, and then the BamHI/EcoRI right arm was cloned into the counterpart site of pWHU1143L to produce pWHU1143R. Subsequently, a thiostrepton resistance gene (*tsr*) was introduced to the BamHI site of this plasmid to give *artG* inactivation plasmid pWHU1143. Likewise, *artB*/*artX* inactivation plasmids pWHU1144/pWHU1145 were constructed using the methods and protocol as mentioned above.

2.2 Generation of *artB, artX, and artB & artX* overexpression vectors.

To generate pWHU1146, the *artB* fragment was amplified with primers *artBexp*-F/R. After NdeI/EcoRI digestion, the fragment was inserted into the corresponding locus of plasmid pIB139 under the control of strong promoter *PermE** to give the *artB* overexpression plasmid pWHU1146. Similarly, pWHU1147 was constructed in consistence with the above method and protocol.

To construct pWHU1149, the *artX* fragment was amplified with primers *artXexp*-F/R and then it was cloned into pJTU968 under the control of *PermE** after the treatment by NdeI/EcoRI to generate pWHU1148. Subsequently, the MfeI-EcoRI engineered fragment containing *PermE** & *artX* was purified from pWHU1148 and it was inserted into the corresponding locus of pWHU1146 adjacent to *PermE** & *art*B region.

3. Supplemental Figures

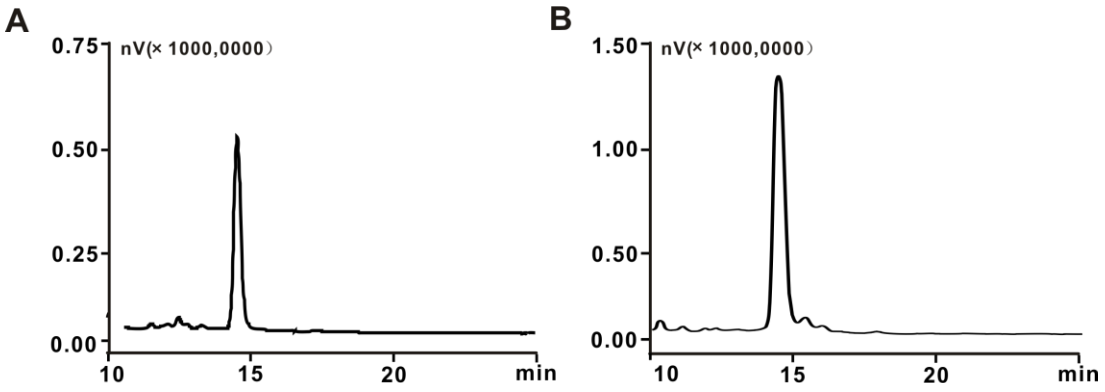

Figure S1. HPLC analysis of aurantimycin A. A: HPLC analysis of aurantimycin A standard; B: HPLC analysis of aurantimycin A production from *S. aurantiacus* JA 4570 wild type.

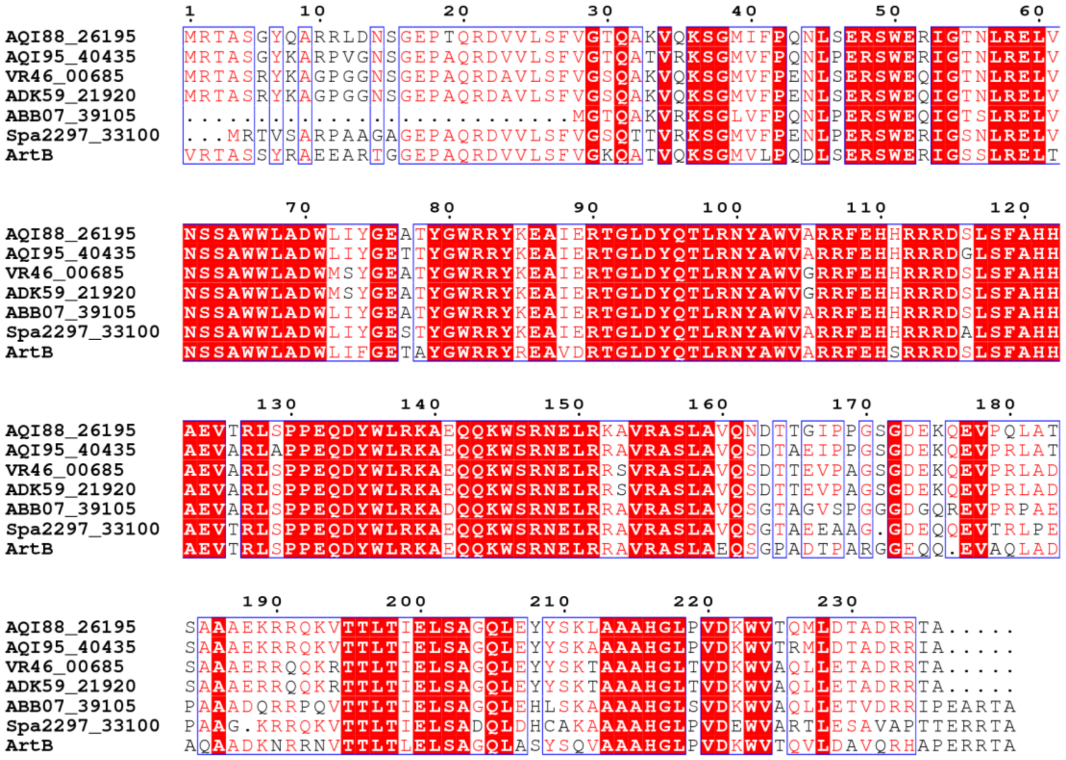

Figure S2. Alignment of ArtB and other regulators from *Streptomyces*. AQI88_26195: the amino acid sequence and secondary structure of AQI88_26195 from *Streptomyces resistomycificus* (Accession no. KUN90566); AQI95_40435: the amino acid sequence and secondary structure of AQI95_40435 from *Streptomyces yokosukanensis* (Accession no. KUM99164); VR46_00685: the amino acid sequence and secondary structure of VR46_00685 from *Streptomyces* sp. NRRL S-444 (Accession no. KJY47946);ADK59_21920: the amino acid sequence of ADK54_22460 ADK59_21920 from *Streptomyces sp.* XY332 (Accession no. KOY55936); ABB07_39105: the amino acid sequence of ABB07_39105 from *Streptomyces incarnatus* (Accession no. AKJ15893); Spa2297_33100: the amino acid sequence of Spa2297_33100 from *Streptomyces parvulus* (Accession no. ANJ11944).

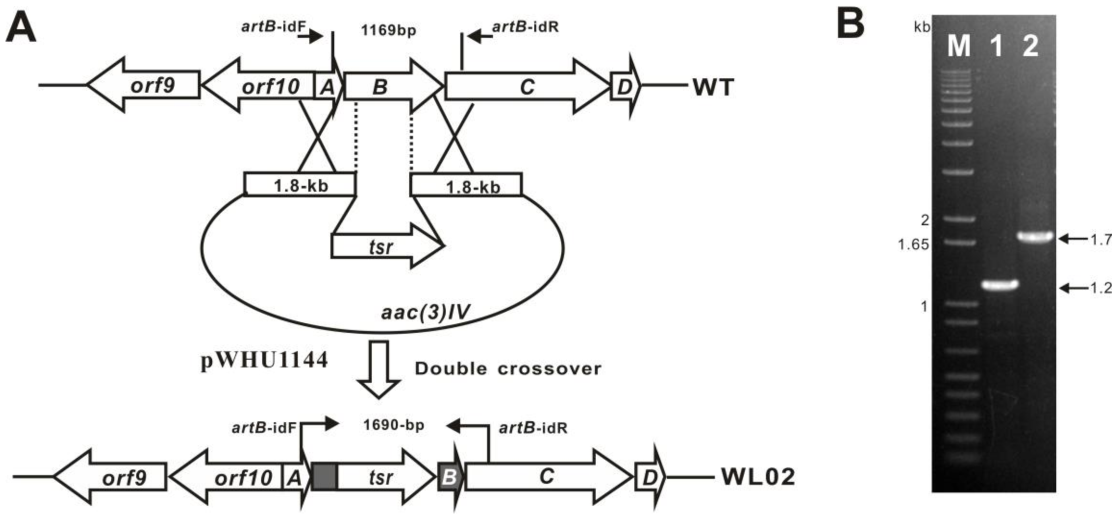

Figure S3. Construction of WL-02 Mutant. **(A)** Schematic representation for construction of WL-02 mutants. **(B)** PCR identification of the WL-02 mutants. M: 1kb plus ladder, 1: Using genomic DNA of JA4570 wild type as template, 2: Using genomic DNA of WL-02 mutants as template.

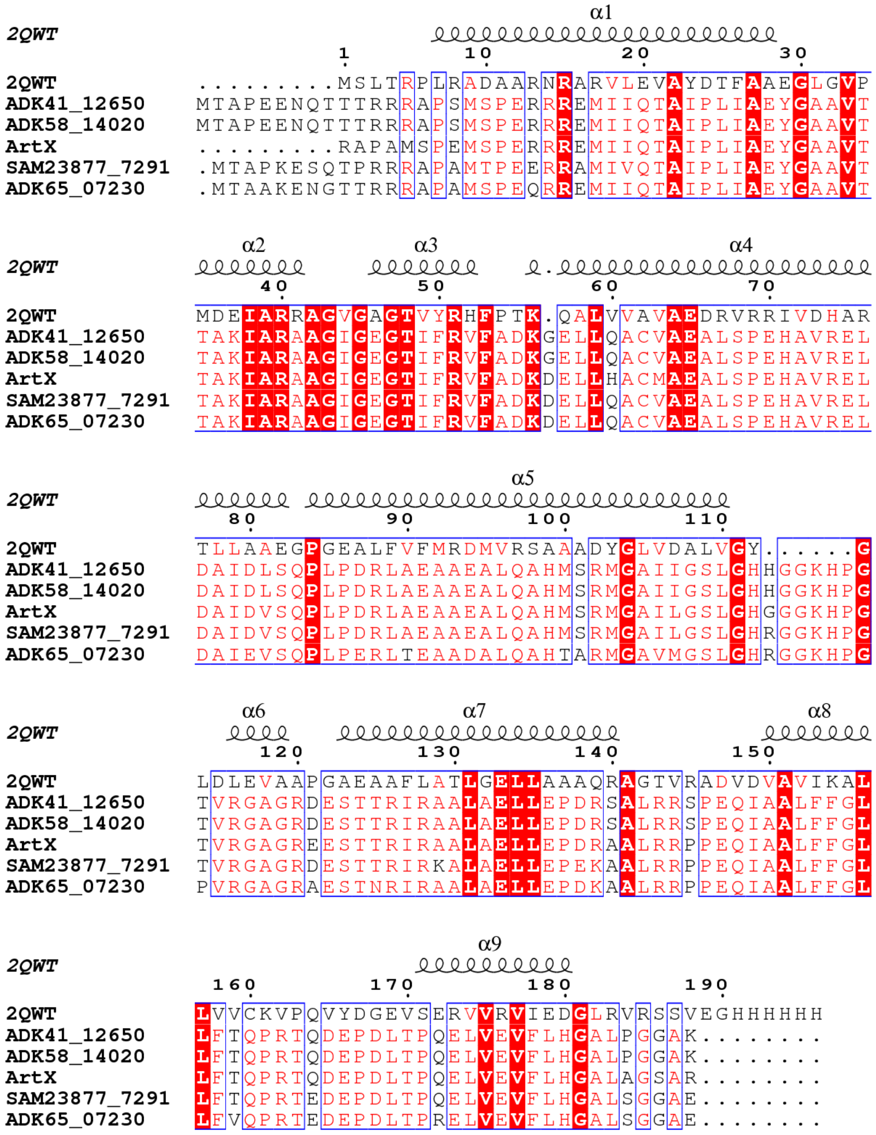

Figure S4. Alignment of TetR regulators and the prediction of ArtX secondary structure. 2QWT: the amino acid sequence and secondary structure of 2QWT from *Mycobacterium vanbaalenii* (Accession no. WP_011778037); ADK41_12650: the amino acid sequence of ADK41_12650 from *Streptomyces caelestis* (Accession no. KOT40573); ADK58_14020: the amino acid sequence of ADK58_14020 from *Streptomyces sp.* XY152 (Accession no. KOV26843). SAM23877_7291: the amino acid sequence of SAM23877_7291 from *Streptomyces ambofaciens* ATCC 23877 (Accession no. AKZ60334); ADK65_07230: the amino acid sequence of ADK65_07230 from *Streptomyces sp.* NRRL B-1140 (Accession no. KOX03342).

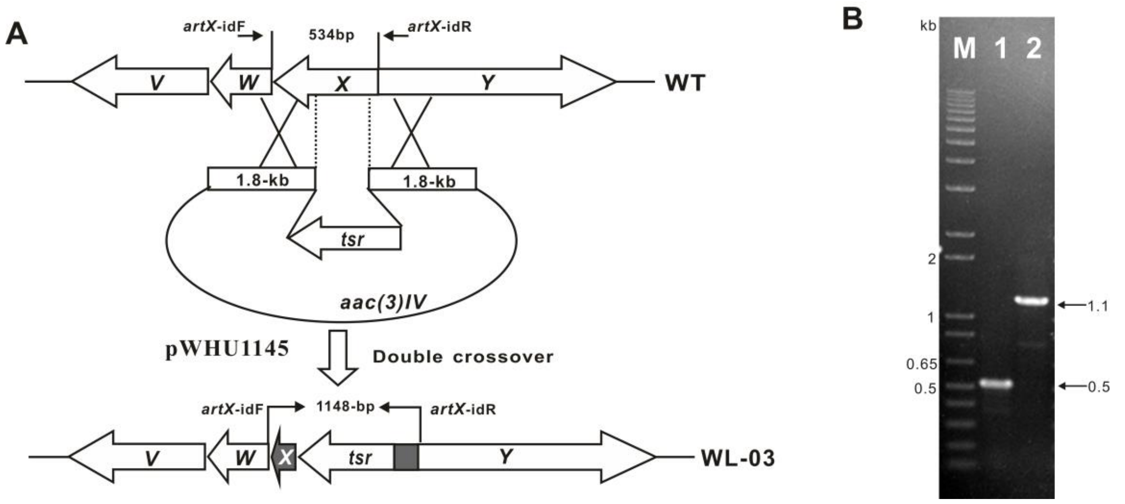

Figure S5. Construction of WL-03 mutants. **(A)** Schematic representation for construction of WL-03 mutants. **(B)** PCR identification of the WL-03 mutants. M: 1kb plus ladder, 1: Using genomic DNA of JA4570 wild type as template, 2: Using genomic DNA of WL-03 mutants as template.

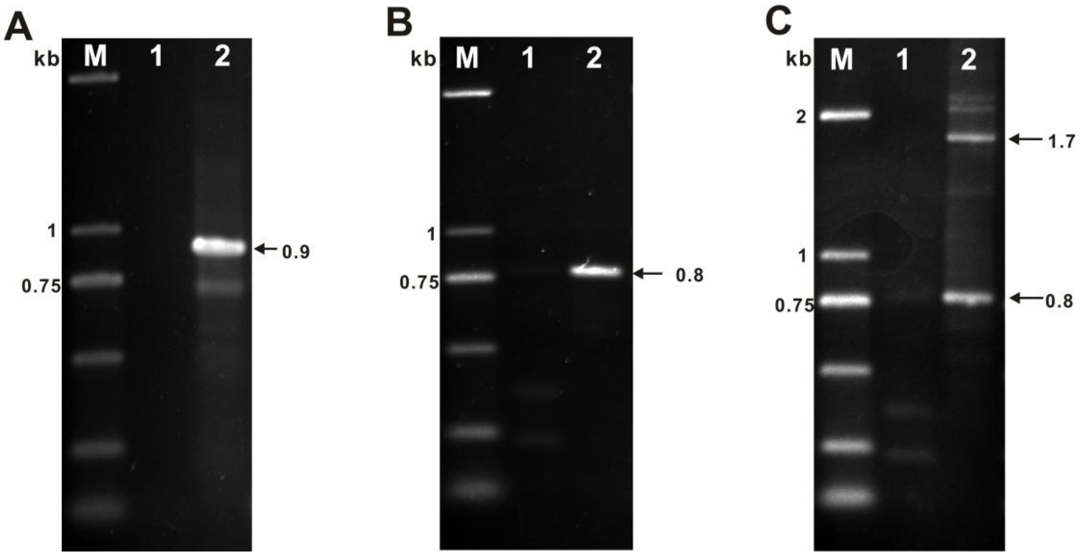

Figure S6. Identification of target gene overexpression strains. **(A)** PCR identification of the *artB* overexpression strains WL-04. M: DM2000, 1, Using genomic DNA of JA4570 wild type as template, 2 Using genomic DNA of WL-04 mutants as template; **(B)** PCR identification of the *artX* overexpression strains WL-05. M: DM2000, 1, Using genomic DNA of JA4570 wild type as template, 2 Using genomic DNA of WL-05 mutants as template; **(A)** PCR identification of the *artB*&*artX* overexpression strains WL-06. M: DM2000, 1, Using genomic DNA of JA4570 wild type as template, 2 Using genomic DNA of WL-06 mutants as template.

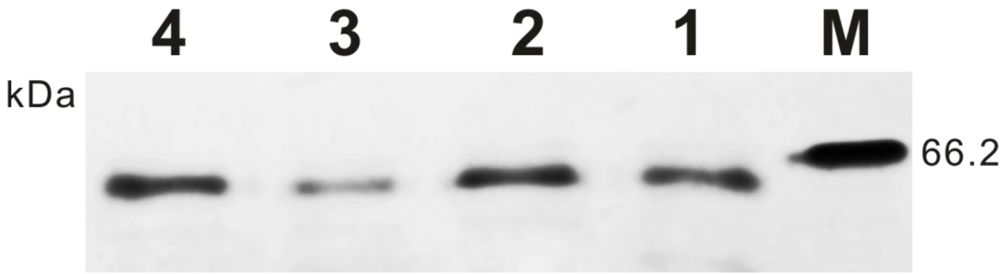

Figure S7. SDS-PAGE analysis of four A domains. M: protein ladder, Unstained Protein Molecular Weight Marker; 1: purified His6-tagged ArtC, 56.9kDa; 2: purified His6-tagged ArtF-A2, 59.6kDa; 3: purified His6-tagged ArtG-A1, 57.3kDa; 4: purified His6-tagged ArtH-A, 57.9kDa.

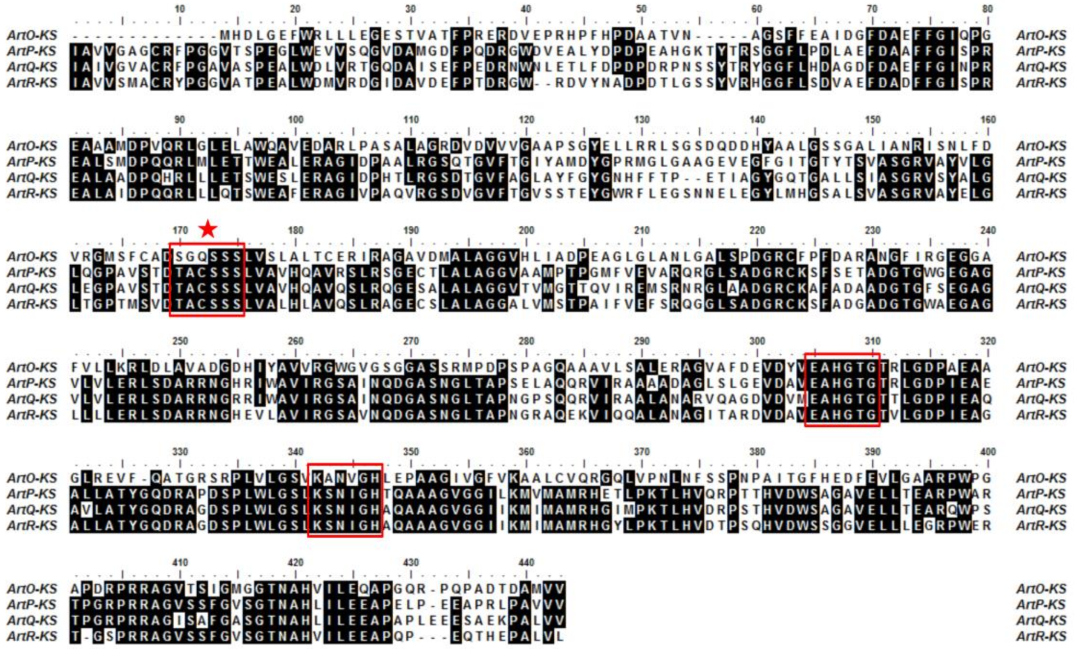

Figure S8. Alignment of four KS domains in PKSs of the *art* cluster. The sequences in rectangular signify the conserved sites for KS domains. The star demonstrates the substitution of the transthioesterification site Cys (C) of ArtP-KS.

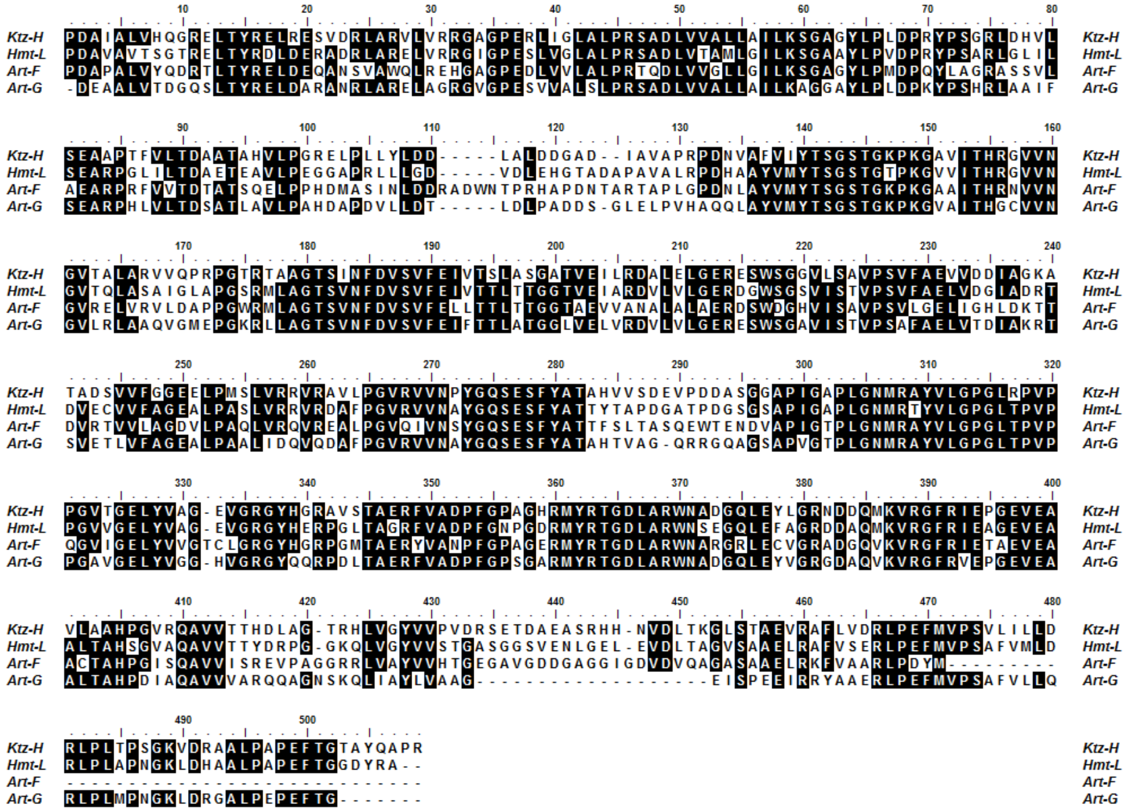

Figure S9. Alignment of A domains activating piperazic acid. Ktz-H: the amino acid sequence of A domain of Ktz-H from *Kutzneria* sp. 744 (Accession no. EU074211); Hmt-L: the amino acid sequence of A domain of Hmt-L from *Streptomyces himastatinicus* ATCC 53653 (Accession no. FR823394).

4. Supplemental References

1. Grafe U, Schlegel R, Ritzau M, Ihn W, Dornberger K, Stengel C, Fleck WF, Gutsche W, Hartl A, Paulus EF: **Aurantimycins, new depsipeptide antibiotics from *Streptomyces aurantiacus* IMET 43917. Production, isolation, structure elucidation, and biological activity.** *J Antibiot (Tokyo)* 1995, **48:**119-125.
2. Paget MS, Chamberlin L, Atrih A, Foster SJ, Buttner MJ: **Evidence that the extracytoplasmic function sigma factor sigmaE is required for normal cell wall structure in *Streptomyces coelicolor* A3(2).** *J Bacteriol* 1999, **181:**204-211.
3. Bierman M, Logan R, O'Brien K, Seno ET, Rao RN, Schoner BE: **Plasmid cloning vectors for the conjugal transfer of DNA from Escherichia coli to *Streptomyces* spp.** *Gene* 1992, **116:**43-49.
4. Del Vecchio F, Petkovic H, Kendrew SG, Low L, Wilkinson B, Lill R, Cortes J, Rudd BA, Staunton J, Leadlay PF: **Active-site residue, domain and module swaps in modular polyketide synthases.** *J Ind Microbiol Biotechnol* 2003, **30:**489-494.
5. Xu H, Zhang Y, Yang J, Mahmud T, Bai L, Deng Z: **Alternative epimerization in C(7)N-aminocyclitol biosynthesis is catalyzed by ValD, a large protein of the vicinal oxygen chelate superfamily.** *Chem Biol* 2009, **16:**567-576.
